# Lessons from the elimination of trachoma in Senegal: implications for high-burden countries in sub-Saharan Africa

**DOI:** 10.1186/s41182-025-00801-w

**Published:** 2025-10-08

**Authors:** Stephen Olaide Aremu, Akyala Ishaku Adamu, Legbel Ikenna Uguru, Abdillahi Abdi Barkhadle

**Affiliations:** 1 Global Health and Infectious Diseases Control Institute, Nasarawa State University, Keffi, Nasarawa Nigeria; 2 Rift Valley University, Jimma, Ethiopia

**Keywords:** Trachoma elimination, SAFE strategy, Community engagement, WASH, Surveillance, Senegal

## Abstract

Senegal’s achievement in eliminating trachoma as a public health problem offers a powerful model for countries still burdened by the disease. This success was driven by a holistic and context-sensitive implementation of the SAFE strategy. The country invested in training health workers to deliver trichiasis surgeries in hard-to-reach areas, ensured high coverage of azithromycin through consistent mass drug administration (MDA), and embedded hygiene promotion in schools and community structures. Importantly, Senegal tailored interventions to regional cultural and epidemiological contexts, enhancing local acceptance and effectiveness. Community ownership, intersectoral collaboration involving education, water, and sanitation ministries, and strong partnerships with international organizations further strengthened outcomes. The use of high-quality data through standardized tools like the Tropical Data platform enabled real-time surveillance, evidence-based programming, and course correction. Even post-elimination, Senegal has maintained vigilance through integrated surveillance and hygiene programs in primary healthcare, ensuring sustained gains and preventing re-emergence. These insights present actionable lessons for other high-burden countries like Nigeria, Ethiopia, and Chad.

Dear Editor,

Senegal, has officially eliminated trachoma as a public health problem. This milestone is not only a national success but also a beacon of hope and a practical model for the 32 countries, particularly in sub-Saharan Africa, still grappling with the burden of this neglected tropical disease [1]. Trachoma remains a public health concern in numerous low-income and underserved regions, with 103 million people globally living in areas that require trachoma-related interventions [2, 3]. In the World Health Organization (WHO) African Region alone, 93 million individuals were estimated to be at risk as of April 2024, accounting for 90% of the global trachoma burden [1]. Despite significant progress, including a 51% reduction in the number of people needing antibiotic treatment in the region between 2014 and 2024, there is still considerable work to be done. The elimination of trachoma in Senegal serves as a case study on the effective implementation of the WHO-recommended SAFE strategy (Surgery, Antibiotics, Facial cleanliness, and Environmental improvement) [1]. Senegal's approach can offer crucial insights into sustainable, scalable public health interventions.

## Political commitment and strategic planning

The Senegalese government demonstrated strong political will and leadership by prioritizing trachoma elimination within its national health agenda. This commitment was evidenced through the development and implementation of a comprehensive national trachoma action plan aligned with the World Health Organization’s SAFE strategy_ Surgery, Antibiotics, Facial cleanliness, and Environmental improvement [1]. Strategic planning ensured that activities were not only well-coordinated but also targeted the most endemic regions with tailored interventions. The allocation of both domestic and international resources, including technical and financial support from partners, such as the WHO, Sightsavers, and The Carter Center, was critical in sustaining momentum toward elimination [4, 5].

## Effective integration of the SAFE strategy

Senegal’s success in eliminating trachoma as a public health problem can be largely attributed to the comprehensive and integrated implementation of the World Health Organization’s SAFE strategy. Unlike piecemeal approaches often observed in other countries, Senegal adopted a well-coordinated, nationwide roll-out of all four components, ensuring that no aspect of the trachoma transmission cycle was left unaddressed. Under the Surgery component, Senegal prioritized the training and deployment of skilled personnel capable of performing trichiasis surgeries at scale. Importantly, these surgical services were not confined to urban hospitals but were extended to hard-to-reach and rural communities where the disease burden was the highest. Mobile surgical teams, supported by logistical coordination, ensured that individuals with advanced disease received timely and effective treatment, thereby preventing blindness and reducing the human cost of the disease [6].

The Antibiotics arm of the strategy was equally robust. Senegal conducted repeated rounds of mass drug administration (MDA) using azithromycin, achieving high-coverage rates and community compliance. This success was due in part to well-organized campaign planning, early community sensitization, and strong logistical frameworks that ensured timely drug delivery and follow-up. Facial cleanliness was embedded in both school-based programs and broader community engagement strategies. Behavior change communication campaigns were crafted to resonate culturally and linguistically with local populations. By targeting children, who are the primary reservoirs of trachoma infection, the country effectively reduced disease transmission within households and communities. Lastly, Senegal invested significantly in environmental improvements. This included widespread construction of latrines, promotion of handwashing, and expansion of clean water access, especially in endemic zones. These efforts addressed the root causes of transmission, creating an enabling environment for lasting elimination. By operationalizing the SAFE strategy in a holistic and inclusive manner, Senegal provides a compelling model for other countries grappling with the persistent burden of trachoma [1, 7].

## Robust surveillance and monitoring systems

Senegal’s commitment to data-driven programming was exemplified by the establishment of robust surveillance systems to track trachoma prevalence and guide intervention strategies. Utilizing standardized tools such as the WHO-supported Tropical Data platform, the country conducted periodic baseline, midterm, and impact assessments across endemic regions. These surveys provided reliable epidemiological data that informed targeted implementation of the SAFE strategy and ensured optimal allocation of resources. The availability of high-quality, geo-referenced data enabled national and regional health teams to make timely, evidence-based decisions and adjust strategies based on emerging trends. Moreover, the surveillance infrastructure strengthened accountability mechanisms and supported the validation process, ensuring that trachoma elimination efforts remained on track and responsive to the needs of at-risk communities [8].

## Community engagement and ownership

A cornerstone of Senegal's success in eliminating trachoma was the deep-rooted involvement of communities at every stage of the intervention process. Community health workers (CHWs), traditional leaders, schoolteachers, women’s groups, and local NGOs were actively engaged in delivering health education, mobilizing households, and facilitating Mass Drug Administration (MDA) campaigns. These actors were not merely implementers but also advocates who translated technical information into culturally relevant messages, thereby boosting public understanding and acceptance. Traditional leaders and religious figures lent their voices to promote behavior change, while schoolteachers integrated hygiene education into daily lessons. This high level of local ownership and participation significantly enhanced program reach, improved adherence to antibiotic treatment and facial cleanliness practices, and ensured the long-term sustainability of interventions post-elimination [9, 10].

## Strong partnership and coordination mechanisms

Senegal’s trachoma elimination success was significantly bolstered by strategic and well-coordinated partnerships with a wide array of stakeholders [1, 2]. The government collaborated closely with international donors, non-governmental organizations (NGOs), academic institutions, and the World Health Organization (WHO). These partnerships provided critical support in the form of technical expertise, funding, personnel training, and operational guidance. NGOs played a pivotal role in community engagement and behavior change communication, while research institutions contributed to disease mapping, surveillance, and impact evaluation. The WHO offered standardized frameworks and validation tools that ensured interventions were aligned with global best practices. This integrated partnership model fostered knowledge sharing, reduced duplication of efforts, and promoted coherence across activities including accelerating progress toward trachoma elimination and strengthening the broader health system in Senegal.

## Cross-sectoral collaboration

Efforts to eliminate trachoma in Senegal extended far beyond the traditional boundaries of the health sector, demonstrating a robust model of inter-sectoral collaboration**.** The government engaged multiple ministries, including those of education, water and sanitation, and rural development, to address the environmental and behavioral determinants of the disease. For instance, the Ministry of Water and Sanitation facilitated the construction of boreholes and latrines in underserved communities, directly supporting the "E" component of the SAFE strategy. Simultaneously, the Ministry of Education integrated hygiene education into school curricula to promote sustained behavioral change among children. This coordinated, multisectoral approach ensured that health outreach was reinforced by improvements in infrastructure and public awareness, creating an enabling environment for effective and lasting trachoma elimination [11, 12]**.**

## Tailored and context-specific interventions

Senegal’s approach to trachoma elimination was marked by a high degree of contextual adaptability, ensuring that interventions were tailored to the unique needs of each region. Rather than adopting a one-size-fits-all model, the program considered local epidemiological patterns, cultural beliefs, and existing infrastructural capacities in planning and implementation [11, 13–16]. For Mass Drug Administration (MDA), a robust supply chain management system was established to prevent drug shortages and minimize the risk of expiry. This involved accurate forecasting based on population data, timely procurement, and phased distribution to regional health facilities. Drugs were stored under recommended conditions and inventory was monitored through a real-time tracking system, enabling rapid redistribution of stock to areas with higher-than-expected demand. Pre-distribution audits and post-MDA reconciliation ensured accountability and minimized wastage.

In regions with high disease burden, intensified MDA and surgical outreach were prioritized. Full geographical coverage for trachomatous trichiasis (TT) case finding and surgical services was systematically documented using standardized reporting templates and geo-referenced maps, confirming that all endemic districts were reached. Cultural norms influencing hygiene practices were addressed through community-specific health education, often delivered by local leaders and community health workers. For remote or nomadic populations, mobile health teams ensured consistent access to services. This flexibility and localization fostered strong community trust, boosted intervention uptake, and ultimately contributed to the overall success of the national trachoma elimination strategy.

## Sustainability and post-elimination surveillance

Even after achieving the World Health Organization’s validation for trachoma elimination, Senegal did not become complacent. Instead, the country proactively instituted robust mechanisms for post-validation surveillance to ensure that the disease does not re-emerge. These surveillance systems were embedded within the routine operations of primary healthcare services, allowing for early detection and rapid response to any signs of resurgence. Additionally, Senegal maintained continuous promotion of Water, Sanitation, and Hygiene (WASH) interventions to sustain behavior change and prevent reinfection. School-based hygiene education programs were also strengthened to instill lifelong healthy practices among children. Moreover, trichiasis surveillance was integrated into community health systems, ensuring that residual or new cases of advanced disease are promptly identified and managed, thereby securing long-term public health gains [11, 14].

### Challenges associated with eliminating trachoma in Senegal

Like many large-scale public health programs, Senegal’s trachoma elimination initiative faced several operational and contextual challenges. One persistent issue was the risk of recurrent infection due to cross-border population movement, particularly in regions bordering countries with ongoing transmission. To address this, cross-border collaborations were established, including synchronized Mass Drug Administration (MDA) campaigns and joint health education activities with neighboring countries. The MDA supply chain also presented difficulties, such as delays in drug delivery linked to late signing of Memoranda of Understanding (MOUs) and the need to manage drugs with approaching expiry dates. These challenges were mitigated through improved forecasting, early procurement planning, and the creation of buffer stocks at regional depots to ensure uninterrupted supply. Conducting full geographical coverage for trachomatous trichiasis (TT) surgery was occasionally hindered by access constraints in remote and hard-to-reach areas, which were overcome by deploying mobile surgical teams and leveraging community volunteers for case finding. Delivery of Facial Cleanliness and Environmental Improvement (F&E) components was at times limited by community reluctance to adopt new hygiene practices and infrastructural gaps such as inadequate water supply. These barriers were addressed through culturally tailored health promotion delivered by trusted local leaders, and by integrating F&E messaging into existing community gatherings. By openly confronting and resolving these challenges, the program strengthened its operational resilience and enhanced the sustainability of trachoma elimination efforts.

### Lessons for high-burden countries

Efforts to eliminate trachoma in countries such as Nigeria, Ethiopia, and Chad must be guided by practical, evidence-based strategies derived from successful case studies like Senegal. One of the foremost lessons is the importance of securing political buy-in. High-level political commitment ensures that trachoma elimination becomes a national priority, catalyzing the mobilization of resources, the alignment of relevant policies, and the creation of structures for effective oversight and accountability. Political will translates to budgetary allocations, multi-sectoral coordination, and an enabling policy environment that can sustain interventions long after donor support wanes. Equally crucial is the need to implement the SAFE strategy holistically. While mass drug administration (MDA) with azithromycin remains a key pillar of trachoma control, countries must look beyond pharmacological interventions. The environmental and behavioral components, specifically facial cleanliness and improved environmental sanitation are vital in breaking the cycle of reinfection. Success hinges on integrating facial hygiene education in schools, ensuring access to water, and improving sanitation infrastructure, particularly in underserved rural communities.

Leveraging community structures plays a pivotal role in achieving program success. Community health workers (CHWs), traditional leaders, religious figures, and local women's groups often command trust and influence at the grassroots level. Mobilizing these actors helps enhance awareness, address cultural barriers, and increase treatment uptake. Involving communities from planning through implementation ensures that interventions are contextually appropriate and culturally sensitive, thereby improving acceptance and sustainability. Strengthening data systems is another lesson of paramount importance. Accurate, timely, and disaggregated data are essential for tracking progress, targeting interventions, and allocating resources efficiently. Surveillance systems must be responsive and capable of identifying hotspots, while also supporting operational research to evaluate intervention outcomes and inform evidence-based adjustments.

In addition, fostering partnerships at global, regional, and local levels is critical. Engagement with the World Health Organization (WHO), international NGOs, academic institutions, and bilateral donors can provide technical support, funding, and strategic direction. Importantly, fostering south-south collaboration allows countries to learn from each other’s experiences, adapt successful models like Senegal’s, and build a collective momentum toward regional elimination.

## Conclusion

Senegal’s elimination of trachoma as a public health problem is a powerful example of what is possible through strategic planning, integrated implementation, community involvement, and sustained investment. As trachoma remains a threat to millions across Africa and beyond, the global community must harness these lessons to accelerate progress in remaining endemic countries. The road to trachoma elimination is challenging but navigable, Senegal has paved the way. We urge policymakers, global health leaders, and donors to study and adapt Senegal’s model to local contexts. With coordinated efforts and the right investments, global trachoma elimination by 2030 remains an achievable goal.

## Recommendations

To accelerate the elimination of trachoma and sustain progress in endemic countries, several strategic actions must be prioritized. One critical step is the establishment of National Elimination Task Forces with multisectoral representation. These task forces should comprise stakeholders from health, education, water, sanitation, finance, and local government sectors. Their role would be to coordinate and oversee national elimination strategies, ensure alignment of efforts across ministries, and provide technical and policy guidance. Such coordinated bodies have been instrumental in countries like Senegal, ensuring cohesive implementation of the SAFE strategy. Another essential component is the allocation of domestic resources to reduce overreliance on donor funding. While international partners can catalyze efforts, long-term sustainability depends on domestic financing. Governments must prioritize trachoma elimination in their national budgets and mobilize both public and private sector investments to support interventions such as mass drug administration, WASH infrastructure development, and behavior change communication.

It is equally important to integrate trachoma control into broader Neglected Tropical Disease (NTD) programs and Universal Health Coverage (UHC) initiatives. This integration enhances efficiency, avoids duplication of efforts, and ensures that trachoma services are part of routine healthcare delivery systems. Embedding trachoma activities within existing community and primary health care structures will also make them more sustainable and cost-effective. Moreover, promoting knowledge sharing through south-south cooperation, particularly through facilitated learning visits to Senegal offers countries an opportunity to understand practical, adaptable components of a successful elimination model. These visits allow stakeholders to see firsthand how intersectoral collaboration, community engagement, and political will can translate into impactful outcomes. Finally, enhancing school health programs with strong hygiene education fosters early adoption of facial cleanliness practices among children and reinforces positive behavior in households. Schools can serve as platforms for health promotion and disease prevention, ensuring that hygiene behaviors are passed on to future generations.

## Data Availability

The datasets generated and analyzed during the current study are not publicly available due to privacy considerations of the participants but are available from the corresponding author upon reasonable request.
